# Genetically predicted gut microbiota mediate the association between plasma lipidomics and primary sclerosing cholangitis

**DOI:** 10.1186/s12876-024-03246-3

**Published:** 2024-05-08

**Authors:** Jie Zhou, Dagang Zhu, Yixin Xu, Chao Chen, Kun Wang

**Affiliations:** 1 Department of General Surgery, Wujin Hospital Affiliated with Jiangsu University, No. 2, Yongning North Road, Changzhou, 213003 Jiangsu Province China; 2 Department of General Surgery, The Wujin Clinical college of Xuzhou Medical University, Changzhou, 213003 China

**Keywords:** Mendelian randomization, Causal relationship, Single nucleotide polymorphism

## Abstract

**Background:**

Primary sclerosing cholangitis (PSC) is a complex disease with pathogenic mechanisms that remain to be elucidated. Previous observational studies with small sample sizes have reported associations between PSC, dyslipidemia, and gut microbiota dysbiosis. However, the causality of these associations is uncertain, and there has been no systematic analysis to date.

**Methods:**

The datasets comprise data on PSC, 179 lipid species, and 412 gut microbiota species. PSC data (*n* = 14,890) were sourced from the International PSC Study Group, while the dataset pertaining to plasma lipidomics originated from a study involving 7174 Finnish individuals. Data on gut microbiota species were derived from the Dutch Microbiome Project study, which conducted a genome-wide association study involving 7738 participants. Furthermore, we employed a two-step Mendelian randomization (MR) analysis to quantify the proportion of the effect of gut microbiota-mediated lipidomics on PSC.

**Results:**

Following a rigorous screening process, our MR analysis revealed a causal relationship between higher levels of gene-predicted Phosphatidylcholine (O-16:1_18:1) (PC O-16:1_18:1) and an increased risk of developing PSC (inverse variance-weighted method, odds ratio (OR) 1.30, 95% confidence interval (CI) 1.03–1.63). There is insufficient evidence to suggest that gene-predicted PSC impacts the levels of PC O-16:1_18:1 (OR 1.01, 95% CI 0.98–1.05). When incorporating gut microbiota data into the analysis, we found that Eubacterium rectale-mediated genetic prediction explains 17.59% of the variance in PC O-16:1_18:1 levels.

**Conclusion:**

Our study revealed a causal association between PC O-16:1_18:1 levels and PSC, with a minor portion of the effect mediated by Eubacterium rectale. This study aims to further explore the pathogenesis of PSC and identify promising therapeutic targets. For patients with PSC who lack effective treatment options, the results are encouraging.

**Supplementary Information:**

The online version contains supplementary material available at 10.1186/s12876-024-03246-3.

## Background

Primary sclerosing cholangitis (PSC) is a rare and chronic cholestatic liver disease characterized by intrahepatic or extrahepatic stricturing, or both, accompanied by bile duct fibrosis [1]. Its prevalence is highest in northern Europe and lower in Asia, with about 16.2 cases per 100,000 people [2]. The causes of PSC remain unknown, and there are limited treatment options, leading to a median survival time of 13.2 years post-diagnosis before liver transplantation or mortality [3].

Recent studies have increasingly focused on dyslipidemia in patients with PSC. Jorgensen et al. highlighted the prevalence of hypercholesterolemia in PSC, which worsens with disease severity [4]. Additionally, Gandelman et al. proposed a disease-specific mechanism for dyslipidemia in PSC, based on a case study linking lipid abnormalities to hepatic impairment [5]. In parallel, studies with relatively small sample sizes (*n* < 30) have also explored lipidomic alterations in patients with PSC compared to healthy individuals [6, 7]. Inevitably, the aforementioned observational studies have limitations such as small sample sizes, residual confounding, and reverse causality, which may hinder causal assessment of the relationship between plasma lipidomics and PSC. For instance, Stokkeland K et al. had shown that administering lipid-lowering medications resulted in beneficial outcomes for PSC patients [8]. Consequently, a contentious debate persists regarding the etiological role of lipid abnormalities in PSC, prompting inquiry into whether these perturbations are foundational to the disease pathology or consequential outcomes thereof.

In recent years, modern and efficient lipidomics technologies have significantly expanded our knowledge of the diversity and breadth of circulating lipids. Lipid species encompass a wide range, including glycerophospholipids, glycerolipids, sphingolipids, and sterols [9]. Genome-wide association studies (GWAS) have transformed our comprehension of the genetic variations influencing lipid levels [10, 11]. Consequently, a study design that minimizes biases is essential to more convincingly establish the causal relationship between lipidomics and PSC.

The intestinal microbiota consists of approximately 4 × 10^13^ commensal bacteria, also known as the “human second genome” [12]. Recently, with the emergence of the “leaky gut” hypothesis and the “PSC microbiome” hypothesis, the gut microbiota has attracted increasing attention as a potential pathogenic factor for PSC [13–15]. Not only that, but changes in blood lipids have been found to be closely related to the abundance of the gut microbiota [16–19]. Consequently, the gut microbiota composition might serve as potential mediators between lipidomics and PSC.

Mendelian randomization (MR) is a potential causal inference method that utilizes genetic variations as instrumental variables to infer the impact of exposure factors on outcomes from observational data [20]. MR can mitigate the effects of unmeasured confounders or biases while leveraging Mendelian inheritance patterns to avoid reverse causation [20]. In this study, we collected recently published summary statistics from GWAS for 149 lipid species, 412 gut microbiota species, and PSC. Through the application of the mediation MR analysis, our primary goal was to unveil the causal relationships among these variables, shed light on the pathogenesis of PSC, and identify promising therapeutic targets.

## Methods

### Study design

The data utilized in our analysis were publicly available and had been approved by the institutional review committees of the respective studies. Hence, no ethical committee review was necessary for this study. Furthermore, all findings generated are detailed in the article and its supplementary materials.

In this study, we explored the causal relationship between plasma lipid species and PSC by bidirectional MR. In addition, the mediating roles played by plasma metabolites were explored. In our study, single nucleotide polymorphisms (SNPs) were defined as instrumental variables (IVs) [21].

### GWAS summary data sources

The data utilized in our study are publicly available, with detailed information provided in Table 1. Specifically, the PSC dataset was obtained from the International PSC Study Group by Sun-Gou Ji et al., focusing on the correlation between PSC quantity and SNPs. This is the largest GWAS dataset on PSC so far, with 2,871 PSC patients and 12,019 controls included [22]. The dataset concerning the plasma lipidomics originated from a study involving 7174 Finnish individuals. This investigation encompassed univariate and multivariate whole-genome analyses, encompassing 179 lipid species (Table S1). Through precise locus mapping and gene prioritization, associations with diseases were explored among 377,277 participants in FinnGen [9]. The data on 412 gut microbiota species came from the Dutch Microbiome Project study. In this research, a GWAS was conducted on a population of 7,738 participants, covering 207 microbial taxa and 205 pathways representing microbial composition and function [23]. It is noteworthy that all GWAS data were sourced from various consortia or organizations, ensuring no sample overlap.

**Table 1:** Details of the genome-wide association studies and datasets used in our analyses

Items	Sample size (cases/controls)	Data sources	PMID	Data Download Link
PSC	14,890 (2,871/12,019)	IPSCSG	27,992,413	https://gwas.mrcieu.ac.uk/; ID: ieu-a-1112
412 Gut Microbiota	7,738	DMP	35,115,690	https://www.ebi.ac.uk/gwas/; Accession numbers GCST90027446-GCST90027857
179 Plasma Lipidome	384,451	-	37,907,536	https://www.ebi.ac.uk/gwas/; Accession numbers GCST90277238 - GCST90277416

PSC, Primary sclerosing cholangitis; IPSCSG, International PSC Study Group; CLSA: Canadian Longitudinal Study on Aging; DMP, Dutch Microbiome Project

### Instrumental variable selection and data harmonization

In our analysis, we incorporated SNPs that exhibited genome-wide significance (*P <* 1 × 10^− 5^). Subsequently, these SNPs were grouped based on linkage disequilibrium (using a window size of 10,000 kb and r^2^ < 0.001). Moreover, palindromic and ambiguous SNPs were omitted from the IVs for the MR analysis [24]. The F statistic was computed by evaluating the variance explained by the SNPs for each exposure, calculated as [(N – K – 1)/K]/ [R^2^/ (1 – R^2^)], where K represents the number of genetic variants and N denotes the sample size. We excluded weak IVs (F-statistics < 10) from the analysis to ensure robustness and reliability of the results [25].

### Statistical analysis

We conducted MR analysis using R software (version 4.2.0, http://www.r-project.org) in conjunction with the “Two-Sample MR” package (version 0.5.6) for precise and comprehensive analysis [26]. Moreover, the online tool PhenoScanner was used to assess all known phenotypes related to the considered genetic instruments in our analyses (http://www.phenoscanner.medschl.cam.ac.uk/).

### Primary analysis

**Fig. 1:**
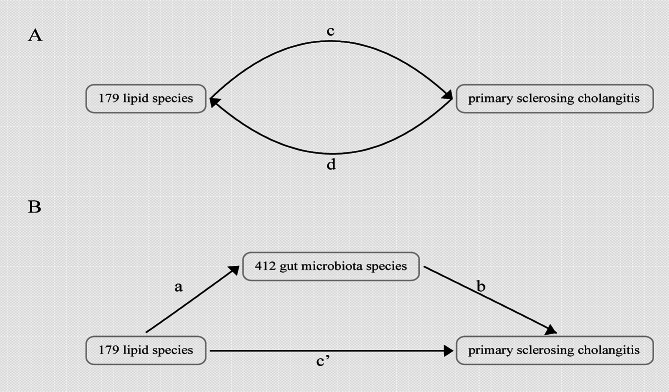
Diagrams illustrating associations examined in this study. (**A**) The total effect between plasma lipidomics and primary sclerosing cholangitis (PSC). c is the total effect using genetically predicted plasma lipidomics as exposure and PSC as outcome. d is the total effect using genetically predicted PSC as exposure and plasma lipidomics as outcome. (**B**) The total effect was decomposed into: (i) indirect effect using a two-step approach (where a is the total effect of plasma lipidomics on gut microbiota species, and b is the effect of gut microbiota species on PSC) and the product method (a × b) and (ii) direct effect (c′ = c – a × b). Proportion mediated was the indirect effect divided by the total effect

Figure 1A presents a schematic overview of our analysis, where we conducted a two-sample bidirectional MR study to explore the reciprocal causation between the plasma lipidomics and PSC. The Inverse variance weighting (IVW) method utilized meta-analysis to combine the Wald ratios of causal effects for each SNP [24, 27]. In addition to IVW, we employed supplementary methods such as Bayesian Weighted MR (BWMR) [28] and Weighted-Median [29] methods. These diverse methodologies, tailored to different validity assumptions, were employed to derive MR estimates. More specifically, IVW relies on the assumption that all SNPs function as valid instrumental variables, ensuring precise estimations. BWMR, designed for causal inference, addresses challenges by considering uncertainties stemming from weak effects due to polygenicity and by detecting outliers using Bayesian weighting to handle violations of the IV assumption caused by pleiotropy. The Weighted Median method demonstrates superior precision, as indicated by its smaller standard deviation compared to MR-Egger analysis. Even in the presence of horizontal pleiotropy, the Weighted Median method offers a consistent estimate, even when 50% of genetic variants are considered invalid instrumental variables [30].

### Mediation analysis

To investigate the potential role of gut microbiota species in mediating the causal relationship between the plasma lipidomics and PSC, we conducted mediation analyses employing a two-step MR design (Fig. 1B). The total effect (c in Fig. 1A) can be decomposed into an indirect effect (a × b in Fig. 1B) and a direct effect (c’ in Fig. 1B) [31]. We calculated the percentage mediated by the mediating effect by dividing the indirect effect by the total effect.

which was designated as the total effect.

### Sensitivity analysis

Due to variations in experimental conditions, selected populations, and SNPs, heterogeneity may exist in two-sample MR analyses, potentially leading to biased estimates of causal effects.

Consequently, this study conducted heterogeneity tests for the primary IVW and MR-Egger methods. Cochrane’s Q value was utilized to assess the heterogeneity of the genetic instruments, with a *P*-value > 0.05 indicating no significant heterogeneity. Furthermore, an underlying assumption of MR analysis is that IVs solely influence the outcome through exposure, necessitating an examination for horizontal pleiotropy between exposure and outcome [32]. In this study, the MR-Egger intercept method was employed to assess the presence of pleiotropy. A *P*-value > 0.05 suggests a minimal or negligible likelihood of pleiotropy in the causal analysis, allowing for its exclusion. Finally, leave-one-out analysis was used to validate the consistency of the results [33].

## Results

**Fig. 2:**
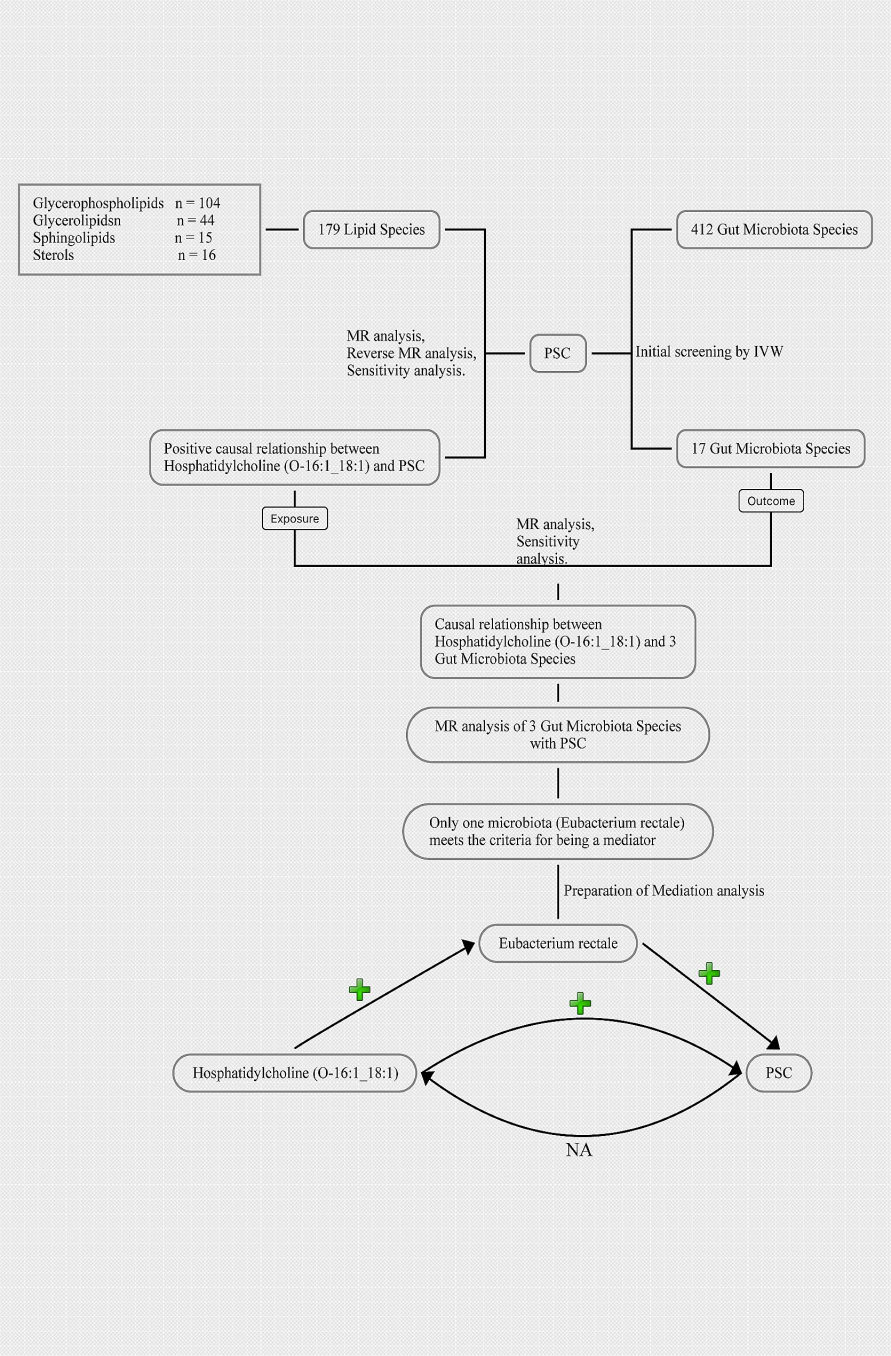
The workflow diagram for this study. PSC, Primary sclerosing cholangitis; IVW, Inverse variance weighting; MR, Mendelian randomization; Eubacterium rectale, k_Bacteria.p_Firmicutes.c_Clostridia.o_Clostridiales.f_Eubacteriaceae.g_Eubacterium.s_Eubacterium_rectale.

The workflow diagram for this study was shown in Fig. 2.

### Association of 179 lipid species and PSC

**Fig. 3:**
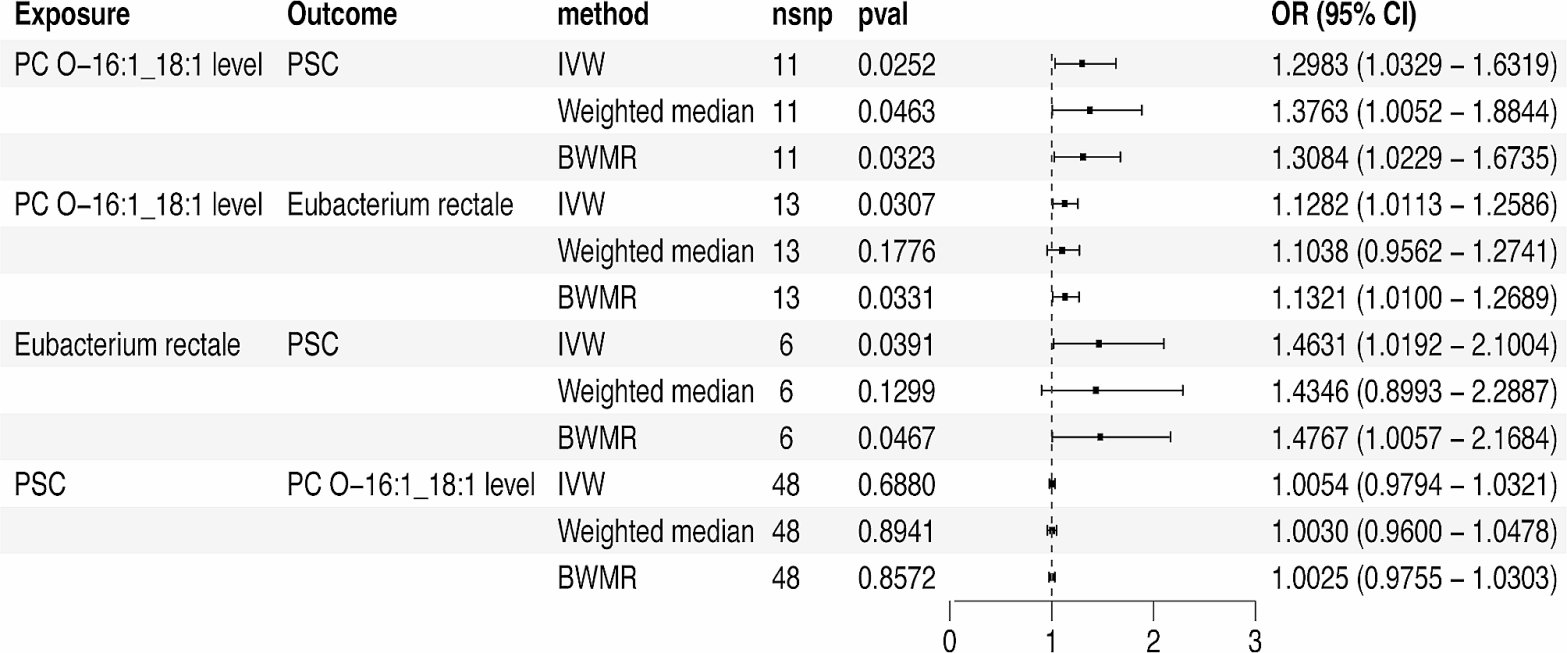
Forest plot to visualize the causal effects of Eubacterium rectale with PC O-16:1_18:1 levels and PSC. PSC, Primary sclerosing cholangitis; PC O-16:1_18:1 levels, Phosphatidylcholine (O-16:1_18:1) levels; Eubacterium rectale, k_Bacteria.p_Firmicutes.c_Clostridia.o_Clostridiales.f_Eubacteriaceae.g_Eubacterium.s_Eubacterium_rectale.

To investigate the genetically predicted causal relationship between the plasma lipidomics and PSC, we treated 179 lipid species as exposures and PSC as the outcome. In this study, the IVW analysis method was used to identify 13 lipid species that may be causally related to PSC, with the exclusion of Phosphatidylcholine (O-16:1_18:2) levels and Triacylglycerol (49:1) levels due to the presence of horizontal pleiotropy (Table S3). Among these 13 lipid species, we only found one that has a positive genetically predicted causal relationship with PSC, which is Phosphatidylcholine (O-16:1_18:1) levels (PC O-16:1_18:1 levels) (IVW odds ratio [OR] per SD increase in PSC = 1.30 [95% CI, 1.03–1.63], *P =* 0.025; Weighted median OR per SD increase in PSC = 1.38 [95% CI, 1.01–1.88], *P =* 0.046; BWMR OR per SD increase in PSC = 1.31 [95% CI, 1.02–1.67], *P =* 0.032) (Fig. 3). Notably, a large number of Triacylglycerol-related lipidomics were found to be negatively correlated with PSC (Table S3).

In our MR analysis, no indication of reverse causality was detected between genetically predicted PC O-16:1_18:1 levels and PSC, with an odds ratio of 1.01 [95% CI, 0.98–1.05, *P =* 0.729] using the IVW method, as illustrated in Fig. 3. The characteristics of SNPs associated with PC O-16:1_18:1 levels and PSC in the bidirectional MR analysis are extensively documented in the supplementary files (Table S4, S5).

### Association of 412 gut microbiota species with PSC

To analyze the data more effectively, we initially investigated the genetically predicted causal relationships between 142 gut microbiota species and PSC by IVW method. Following preliminary screening, a total of 17 gut microbiota species have been identified as potentially causally linked to PSC based on genetically predicted associations (Table S6), pending further scrutiny for heterogeneity and pleiotropy.

### Association of PC O-16:1_18:1 levels with 17 gut microbiota species

In the aforementioned two-sample MR analysis, we have identified the genetically predicted causal relationship between PC O-16:1_18:1 levels and PSC, as well as preliminarily screened a total of 17 gut microbiota species that may potentially be causally related to PSC.

In the upcoming analysis, we will further explore the genetically predicated causal relationships between PC O-16:1_18:1 levels and the 17 gut microbiota species.

Following an initial screening using IVW, potential causal relationships were identified between PC O-16:1_18:1 levels and 3 gut microbiota species. After a comprehensive analysis, it was revealed that PC O-16:1_18:1 levels exhibit a negative causal relationship with FERMENTATION.PWY.mixed.acid.fermentation, while showing a positive causal association with LACTOSECAT.PWY.lactose.and.galactose.degradation.I and k_Bacteria.p_Firmicutes.c_Clostridia.o_Clostridiales.f_Eubacteriaceae.g_Eubacterium.s_Eubacterium_rectale (abbreviated as Eubacterium rectale), with no evidence of horizontal pleiotropy and heterogeneity (Table S7).

### Association of 3 gut microbiota species with PSC

After rigorous screening, a total of 3 gut microbiota species have been included in the candidate list. In the subsequent work, we will focus on analyzing the genetically predicated causal relationships between these 3 gut microbiota species and PSC (Table S8).

The gut microbiota species FERMENTATION.PWY.mixed.acid.fermentation and LACTOSECAT.PWY.lactose.and.galactose.degradation.I, acting as mediators, exhibit opposing correlations between PC O-16:1_18:1 levels and PSC, hence excluded from the study.

Ultimately, we confirmed a positive genetically predicated causal relationship between Eubacterium rectale and PSC through MR analysis (IVW OR per SD increase in PSC = 1.46 [95% CI, 1.02–2.10], *P =* 3.91E-02; BWMR OR per SD increase in PSC = 1.48 [95% CI, 1.01–2.17], *P =* 4.67E-02), successfully passing tests for heterogeneity, horizontal pleiotropy, and sensitivity analysis (Fig. 3). Although Weighted median method failed to yield positive results, the overall direction was consistent.

### Proportion of the association between PC O-16:1_18:1 levels and PSC mediated by Eubacterium rectale

**Fig. 4:**
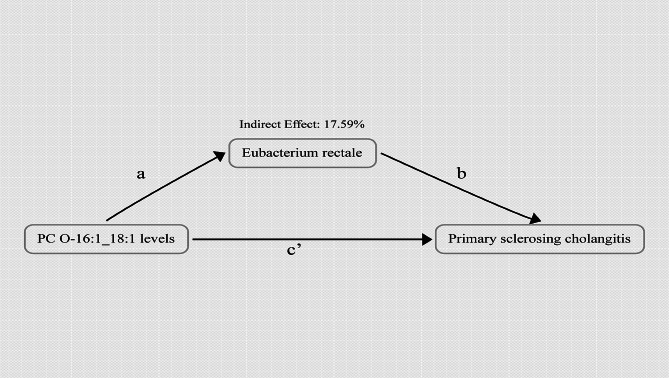
Schematic diagram of the Eubacterium rectale mediation effect. PSC, Primary sclerosing cholangitis; PC O-16:1_18:1 levels, Phosphatidylcholine (O-16:1_18:1) levels Eubacterium rectale, k_Bacteria.p_Firmicutes.c_Clostridia.o_Clostridiales.f_Eubacteriaceae.g_Eubacterium.s_Eubacterium_rectale.

In summary, we have ultimately discovered that Eubacterium rectale can act as a mediator in the pathway from PC O-16:1_18:1 levels to PSC. We observed an association between elevated PC O-16:1_18:1 levels and increased Eubacterium rectale (Table S9), which, in turn, were correlated with an elevated risk of PSC (Table S10). As illustrated in Fig. 1B, our study demonstrated that the indirect effect was 0.05, constituting 17.59% of the heightened risk of PSC associated with PC O-16:1_18:1 levels, while the direct effect was 0.22 (Fig. 4).

### Sensitivity analysis

In the above analysis, we utilized the online tool PhenoScanner to analyze each SNP and found no association between them and the outcome under investigation. Simultaneously, a “leave-one-out” approach was utilized for sensitivity analysis to explore whether specific SNPs influenced the causal relationships. The findings revealed that systematically excluding each SNP did not lead to significant changes in the model’s effect estimates or qualitative conclusions (Supplementary Figure S1, S2, S3, S4). In the MR analysis conducted, tests for heterogeneity and horizontal pleiotropy were performed, with parameters failing to meet the three key assumptions being excluded, ensuring the high credibility of the study results (Table S11).

## Discussion

Most lipids detected in human serum or plasma remain stable and are well correlated with the liver lipidome [34]. Thus, the circulating lipidome is an attractive source of biomarkers for hepatobiliary diseases [35–37]. Previously, studies with relatively limited sample sizes (*n* < 30) have investigated lipidomic changes in patients with PSC compared to healthy individuals [6, 7, 38], but the results were limited by the influence of various confounding factors. Based on our current understanding, this study represents the first comprehensive assessment of the causal relationship between plasma lipidomes and PSC. Previous research has already unveiled the close connections among gut microbiota, PSC, and plasma lipidomes [13–19]. However, existing evidence is confined to observational studies, susceptible to various confounding factors. Leveraging existing GWAS data, this study innovatively employs MR analysis to link these three elements, aiming to demonstrate the causal relationship between PSC and plasma lipidomes. By incorporating GWAS data on gut microbiota, the study explores their potential mediating role.

In this study, our findings suggest that genetically predicted PC O-16:1_18:1 levels were associated with an increased risk of PSC (30% increased risk of PSC for every 1 SD increase in PC O-16:1_18:1 levels), and 17.59% of this effect was mediated through Eubacterium rectale levels in the gut. Furthermore, the MR analyses carried out in this study revealed no indications of horizontal pleiotropy or heterogeneity, and sensitivity analyses also affirmed the robustness of the findings.

PSC is closely related to the intestine. With the emergence of the “leaky gut” hypothesis and the “PSC microbiome” hypothesis, the gut microbiota as a potential pathogenic factor for PSC has received increasing attention [13, 15, 16]. In this study, we identified that the gut microbe Eubacterium rectale can act as a mediator to facilitate the occurrence and progression of PSC by mediating PC O-16:1_18:1. Similarly, Alfonso et al. discovered a positive correlation between plasma phosphatidylcholine and Eubacterium rectale through multi-omics analysis, which aligns with our findings [39]. Furthermore, Wolfgang et al. proposed the discovery of a novel pathway for phosphatidylcholine to be transported from endogenous sources through mucosal cell gaps and across tight junction barrier to the intestinal lumen surface [40]. Phosphatidylcholine in intestinal mucus binds to membrane-bound mucin 3, which is then transferred to mucin 2 to establish a hydrophobic mucus barrier against the colonic luminal microbial community, playing a crucial role in maintaining intestinal microbial homeostasis [40]. However, in the case of tight junction disruption, plasma phosphatidylcholine cannot be transferred to intestinal mucus, leading to elevated levels in the blood and decreased levels in intestinal mucus. This imbalance results in gut dysbiosis, toxin absorption, and the induction of ulcerative colitis [41–43]. Approximately 5% of ulcerative colitis patients progress to PSC, explaining why the majority of PSC patients (70%) also have ulcerative colitis [1].

Choline and its derivatives are not only essential components of cell membranes but also play a crucial role in the transport and metabolism of lipid cholesterol [44]. Furthermore, abnormalities in phospholipid metabolism may impact various biological processes, such as inflammation [45]. In this study, we found that genetic predicted plasma PC O-16:1_18:1 levels have a causal relationship with PSC. In line with the serum metabolomics analysis of a small sample population conducted by Jesus M et al., elevated levels of phosphatidylcholine were observed in the serum of patients with PSC [6]. Interestingly, as phosphatidylcholine serves as a crucial component of bile, a separate study reported a decrease in phosphatidylcholine levels in the bile of PSC patients [46]. The biliary epithelium, akin to other mucosal surfaces, is regarded as a protective surface that secretes mucus to maintain its integrity [47]. It employs phosphatidylcholine bound to mucins produced locally to preserve its hydrophobic nature [48]. Likewise, when tight junction is compromised, the inability of plasma phosphatidylcholine to transfer to biliary mucus results in reduced phosphatidylcholine levels within the bile ducts, consequently triggering the onset of local chronic inflammation [40, 49]. This may be one of the reasons for the direct effect.

This study has several limitations. Firstly, the threshold for significant genome-wide SNPs is typically set at *P* < 5 × 10 − 8. However, in this study, a threshold of *P* < 1 × 10 − 5 was used, which may increase the likelihood of discovering statistically significant SNPs but also raise the risk of false positives. Secondly, while all the analytical results of this study meet the three major assumptions of MR analysis, the overall statistical significance is relatively weak. Additionally, differences in lipidomics and microbiomics between Finnish and Dutch populations may introduce confounding effects related to regional and dietary factors. Dietary habits vary across regions and can influence the composition of the gut microbiota, thereby affecting the associations between plasma lipids and gut bacteria [50, 51]. PSC, as a rare disease, also exhibits variations in incidence rates across different regions [52]. Biases resulting from population differences could potentially impact the results. Finally, the findings of this study warrant validation using larger sample sizes.

## Conclusion

In conclusion, our study revealed a causal association between PC O-16:1_18:1 levels and PSC, with a minor portion of the effect mediated by Eubacterium rectale. The underlying mechanisms may involve the transport pathway of PC mediated by tight junctions in biliary and intestinal epithelial cells. This study aims to further explore the pathogenesis of PSC and identify promising therapeutic targets. For patients with PSC who lack effective treatment options, the results are encouraging.

### Electronic supplementary material

Below is the link to the electronic supplementary material.

Supplementary Material 1

Supplementary Material 2

Supplementary Material 3

Supplementary Material 4

Supplementary Material 5

## Data Availability

Data are available from the corresponding author upon reasonable request.

## Abbreviations

PSC: Primary Sclerosing Cholangitis
MR: Mendelian Randomization
PC O-16:1_18:1: Phosphatidylcholine (O-16:1_18:1)
OR: Odds Ratio
CI: Confidence Interval
GWAS: Genome-Wide Association Studies
SNPs: Single Nucleotide Polymorphisms
IVs: Instrumental Variables
IVW: Inverse Variance Weighting
BWMR: Bayesian Weighted Mendelian Randomization
k_Bacteria.p_Firmicutes.c_Clostridia.o_Clostridiales.f_Eubacteriaceae.g_Eubacterium.s_Eubacterium_rectale: Eubacterium rectale
