# Supplementary figures and images for: Genetically predicted gut microbiota mediate the association between plasma lipidomics and primary sclerosing cholangitis
Source: BMC Gastroenterol. 2024 May 8;24:158. doi: 10.1186/s12876-024-03246-3 (PMC11080140; doi:10.1186/s12876-024-03246-3)

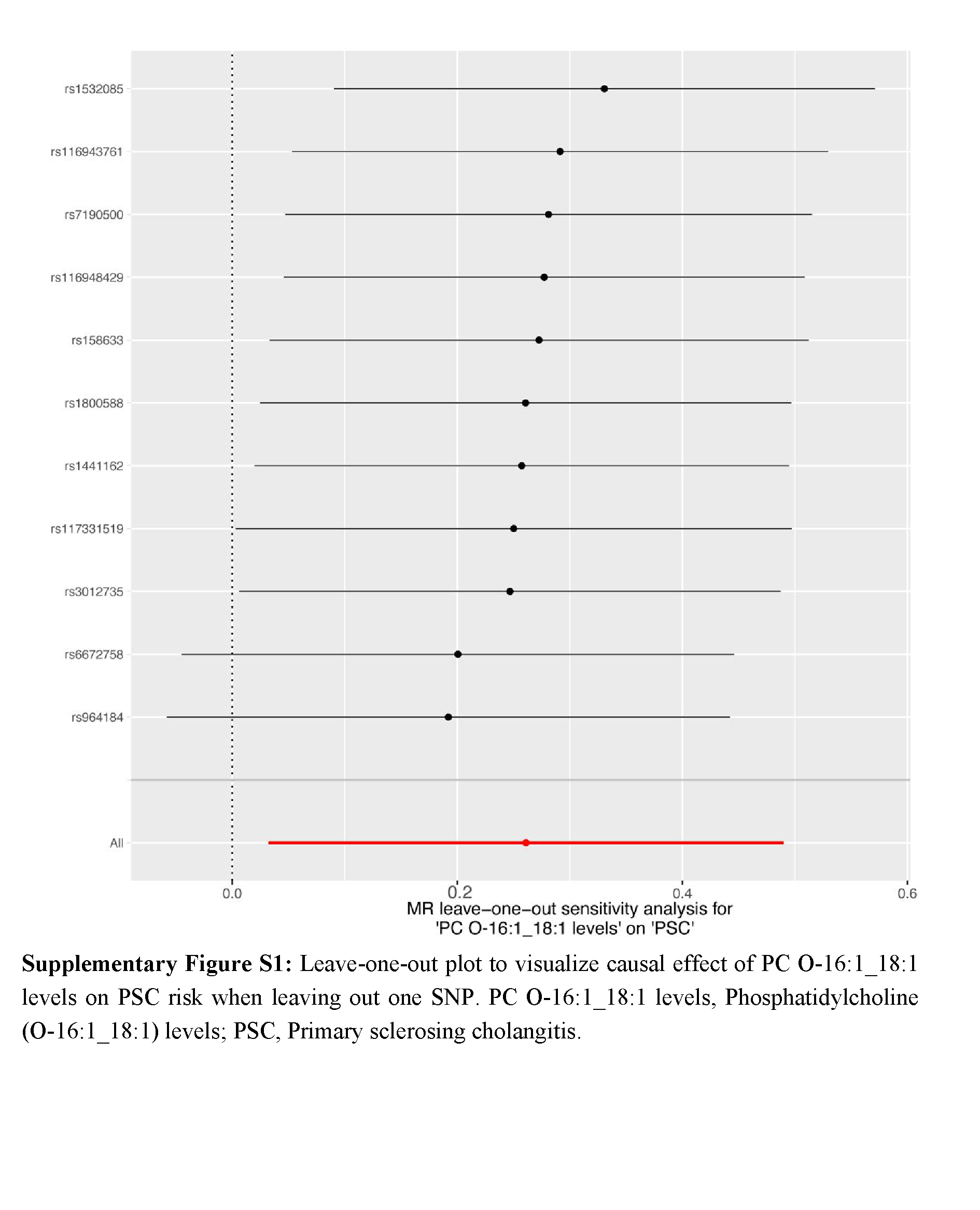

Supplement: Supplementary file 1 — Supplementary Material 1 [file 12876_2024_3246_MOESM1_ESM.tif]

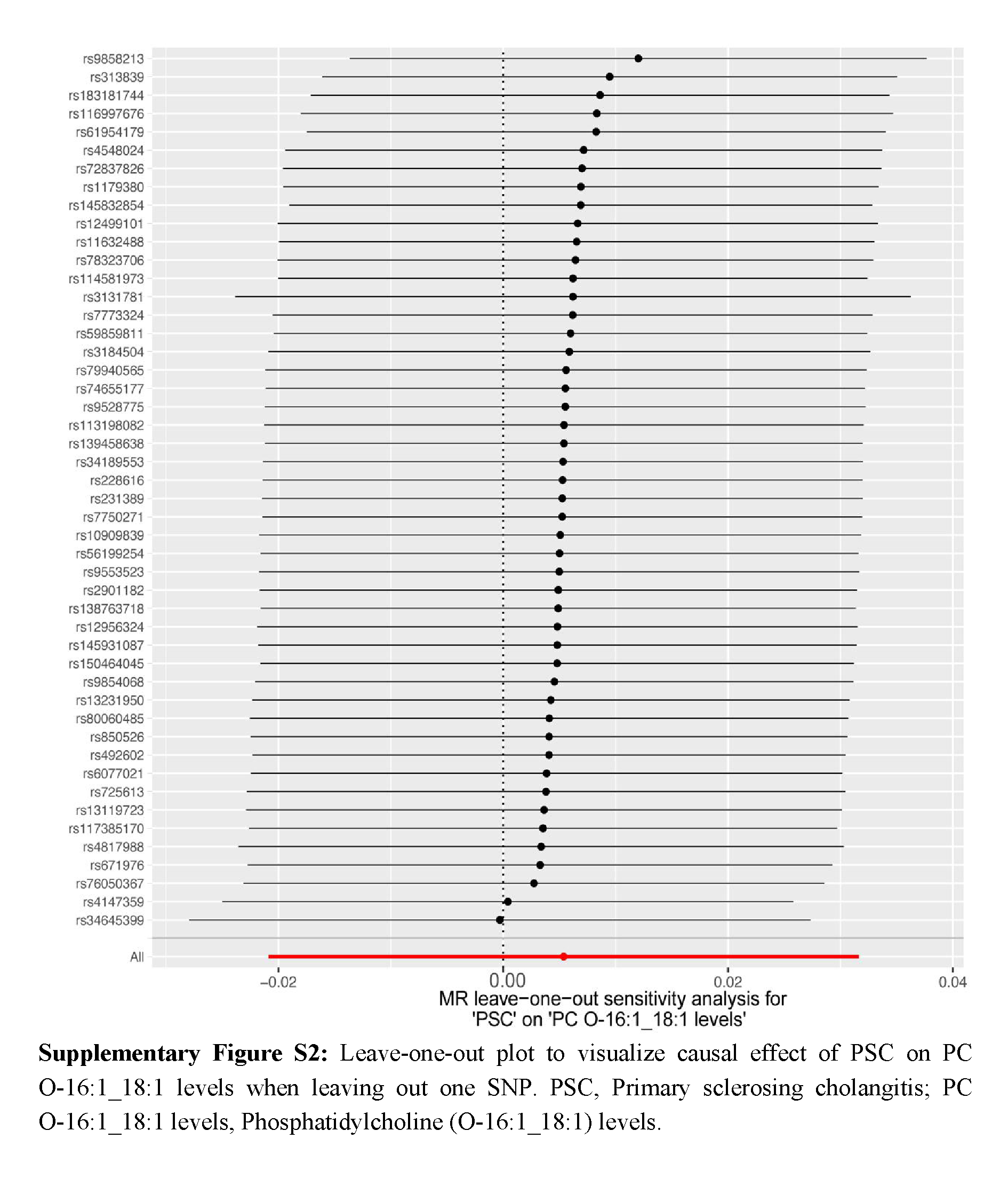

Supplement: Supplementary file 2 — Supplementary Material 2 [file 12876_2024_3246_MOESM2_ESM.tif]

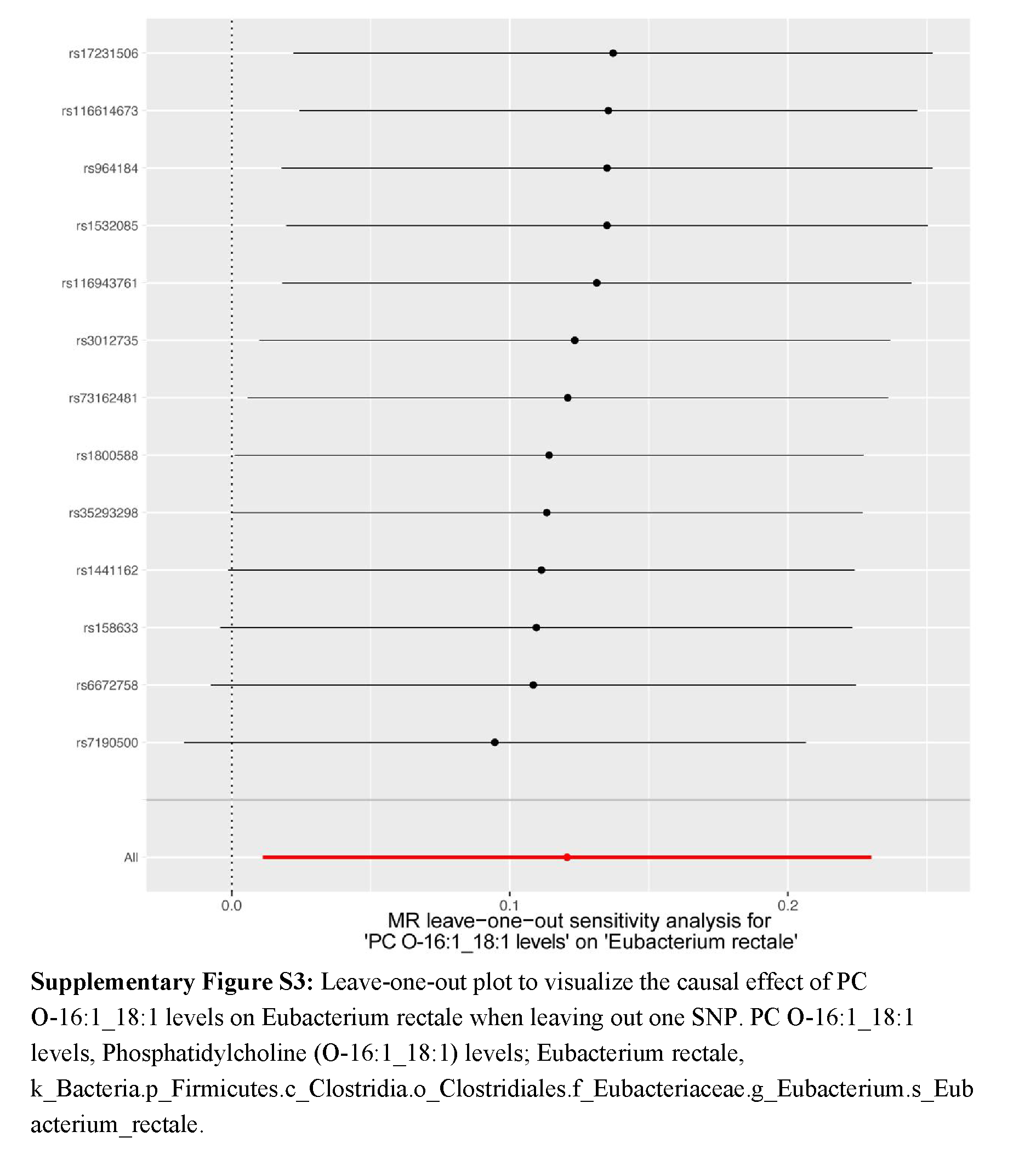

Supplement: Supplementary file 3 — Supplementary Material 3 [file 12876_2024_3246_MOESM3_ESM.tif]

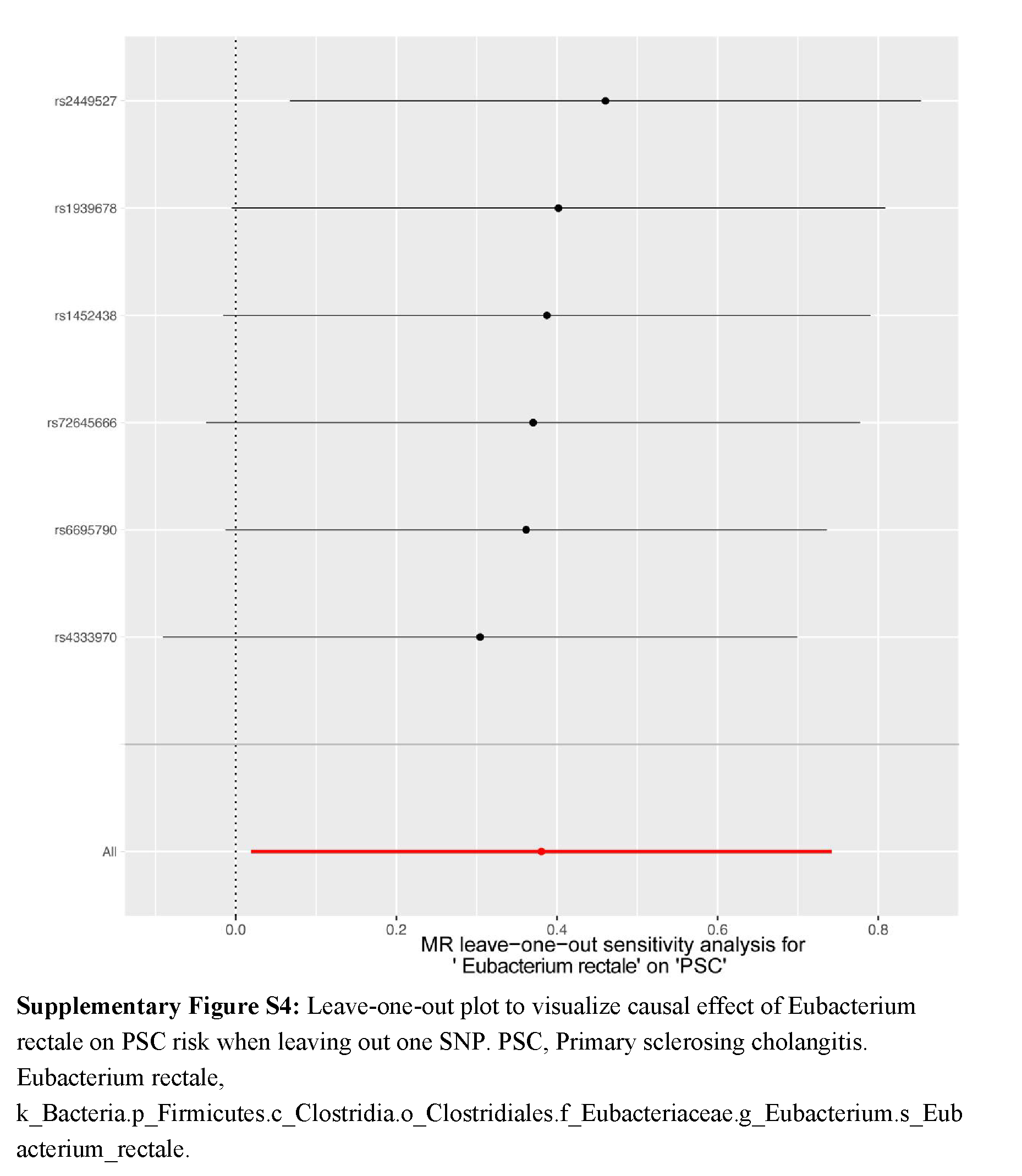

Supplement: Supplementary file 4 — Supplementary Material 4 [file 12876_2024_3246_MOESM4_ESM.tif]
